# Low Repair Capacity of DNA Double-Strand Breaks Induced by Laser-Driven Ultrashort Electron Beams in Cancer Cells

**DOI:** 10.3390/ijms21249488

**Published:** 2020-12-14

**Authors:** Nelly Babayan, Natalia Vorobyeva, Bagrat Grigoryan, Anna Grekhova, Margarita Pustovalova, Sofya Rodneva, Yuriy Fedotov, Gohar Tsakanova, Rouben Aroutiounian, Andreyan Osipov

**Affiliations:** 1 Institute of Molecular Biology NASRA, 7 Hasratyan, Yerevan 0014, Armenia; n_babayan@mb.sci.am (N.B.); g_tsakanova@mb.sci.am (G.T.); 2 Faculty of Biology, Yerevan State University, 1 Manoogian, Yerevan 0025, Armenia; rouben_a@hotmail.com; 3 State Research Center—Burnasyan Federal Medical Biophysical Center of Federal Medical Biological Agency, 46 Zhivopisnaya, 123182 Moscow, Russia; nuv.rad@mail.ru (N.V.); sontyaga@yandex.ru (S.R.); ufedotov456@gmail.com (Y.F.); 4 Semenov Institute of Chemical Physics, Russian Academy of Sciences, 4 Kosygina, 119991 Moscow, Russia; 5 CANDLE Synchrotron Research Institute, 31 Acharyan, Yerevan 0040, Armenia; grigory@asls.candle.am; 6 Emanuel Institute for Biochemical Physics, Russian Academy of Sciences, 4 Kosygina, 119991 Moscow, Russia; annagrekhova1@gmail.com; 7 Moscow Institute of Physics and Technology, 9 Institutskiy per., Dolgoprudny, 141700 Moscow, Russia; mvpustovalova@gmail.com

**Keywords:** laser-driven accelerators, ionizing radiation, ultrashort pulsed electron beam, DNA double-strand breaks, γH2AX, 53BP1, cancer cells

## Abstract

Laser-driven accelerators allow to generate ultrashort (from femto- to picoseconds) high peak dose-rate (up to tens of GGy/s) accelerated particle beams. However, the radiobiological effects of ultrashort pulsed irradiation are still poorly studied. The aim of this work was to compare the formation and elimination of γH2AX and 53BP1 foci (well known markers for DNA double-strand breaks (DSBs)) in Hela cells exposed to ultrashort pulsed electron beams generated by Advanced Research Electron Accelerator Laboratory (AREAL) accelerator (electron energy 3.6 MeV, pulse duration 450 fs, pulse repetition rates 2 or 20 Hz) and quasi-continuous radiation generated by Varian accelerator (electron energy 4 MeV) at doses of 250–1000 mGy. Additionally, a study on the dose–response relationships of changes in the number of residual γH2AX foci in HeLa and A549 cells 24 h after irradiation at doses of 500–10,000 mGy were performed. We found no statistically significant differences in γH2AX and 53BP1 foci yields at 1 h after exposure to 2 Hz ultrashort pulse vs. quasi-continuous radiations. In contrast, 20 Hz ultrashort pulse irradiation resulted in 1.27-fold higher foci yields as compared to the quasi-continuous one. After 24 h of pulse irradiation at doses of 500–10,000 mGy the number of residual γH2AX foci in Hela and A549 cells was 1.7–2.9 times higher compared to that of quasi-continuous irradiation. Overall, the obtained results suggest the slower repair rate for DSBs induced by ultrashort pulse irradiation in comparison to DSBs induced by quasi-continuous irradiation.

## 1. Introduction

In recent years, laser-generated particles’ acceleration technologies have been actively developed. Laser-generated particle beams are characterized by ultrashort duration (from femtoseconds to picoseconds), high peak dose-rate (up to tens of GGy/s during pulse), monoenergetic spectral profile and low side divergence [1,2,3]. Currently, the application of laser-generated accelerators in clinical practice gained attention [4,5,6]. The possibility of generating of directed ultrashort pulses allows the highly precise local influence on solid tumors without affecting nearby healthy tissues [4,5,6]. At the same time, the duration of irradiation by single ultrashort pulse is far lower than the half-life (*t*_1/2_) of free radicals (e.g., hydroxyl radicals *t_1/2_* is ~1 ns [7]), suggesting the appearance of previously unexplored physicochemical processes, which, in turn, can affect the radiobiological effectiveness of pulsed irradiation.

From this perspective, the impact of ultrashort pulsed irradiation on DNA and particularly on one of the most deleterious lesions, namely double strand breaks (DSBs) formation on both qualitative and quantitative levels is of high interest. It is widely accepted that DSBs are the main trigger for the initiation of cellular processes in response to ionizing radiation [8,9]. About 80% of irradiation-induced DSBs are repaired by a relatively fast (up to 4–6 h), but error-prone mechanism of nonhomologous end joining, which often results in microdeletions and chromosomal aberrations [10,11]. Inability to repair of DSBs leads to cells death [12,13].

The aim of the current study was the investigation of the formation and repair kinetics of DSBs, induced by ultrashort pulsed and quasi-continuous electron beam irradiation in human cancer cells.

The immunofluorescence imaging of DNA repair proteins foci has been used for the quantitative study of DSBs formation. Once stained, the complex of dynamic microstructures, formed for DSBs repair and consisting of a set of proteins in many (up to few thousands) copies, can be seen as bright points called foci [14]. The most known DSB markers are phosphorylated H2AX (γH2AX) histone and 53BP1 protein foci [15,16,17,18]. H2AX phosphorylation, mediated by ATM, ATR and DNA-PK sensor kinases, arises in response to DSBs formation and presents the evidence of their recognition [19]. 53BP1 is a p53 binding protein, which is a well known promoter of the DSB repair [20].

Human cervix carcinoma HeLa and human lung carcinoma A549 cell lines were used in this study as the common experimental models for studying various biological effects [21,22].

## 2. Results

### 2.1. Dose-Responses for γH2AX and 53BP1 Foci

Comparative analysis of γH2AX and 53BP1 foci yields was performed at 1 h post-irradiation and 0.25–1.0 Gy doses, following cells the irradiation on Advanced Research Electron Accelerator Laboratory (AREAL) and Varian accelerators. Cell irradiation on the AREAL accelerator was carried out using different pulse repetition and dose-rates (2, 20 Hz and 1.2, 11.7 Gy/min, respectively), while irradiation on Varian machine was done at dose-rate of 5.6 Gy/min.

**Figure 1:**
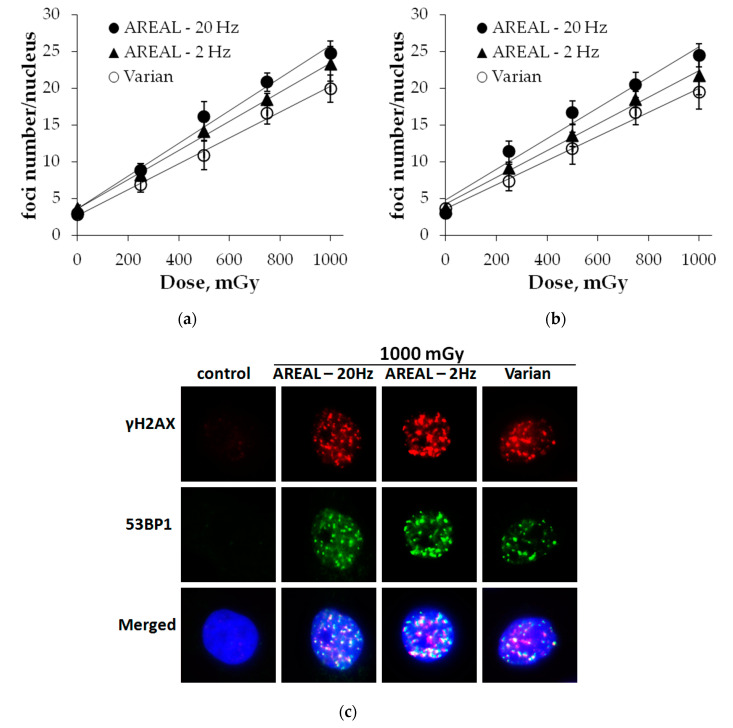
Dose-dependent changes in the γH2AX (**a**) and 53BP1 (**b**) foci numbers in HeLa cells at 1 h post-irradiation by Advanced Research Electron Accelerator Laboratory (AREAL) (ultrashort pulsed 3.6 MeV electron beams) and Varian (quasi-continuous 4.0 MeV electron beams) accelerators; (**c**) representative images of γH2AX and 53BP1 foci in cell nuclei. Nuclei were counter-stained with 4′,6-diamidino-2-phenylindole (DAPI), shown in blue. γH2AX and 53BP1 foci are shown in red and green, respectively. Mean values derived from at least three independent experiments are shown. Error bars show SE.

Dose-dependent changes in γH2AX foci numbers are presented in Figure 1a. It has been shown that following irradiation using the AREAL accelerator at a 20 Hz repetition rate, a dose-dependent increase in foci numbers was described with a high fit (R^2^ = 0.99, *p* < 0.001) by y = 3.57 + 22.29 × x linear equation, where y is an average of foci numbers in a cell nucleus and x is an irradiation dose (Gy). Lowering the repetition rate of irradiation up to 2 Hz resulted in y = 3.62 + 19.82 × x (R^2^ = 0.99, *p* < 0.001) equation, where y is an average of foci numbers in a cell nucleus and x is an irradiation dose (Gy). In a linear equation (y = a + b × x), describing a dose-dependent relationship, the slope coefficient b reflects the increase in the effect per dose unit. The comparison of the slope coefficients of two different dose–response curves, obtained by different exposure types, reflects for how many fold one of them is more expressed compared to the other. After 2 Hz irradiation, the slope coefficient was found to be about 1.13 times lower with respect to 20 Hz, however, this difference was not statistically significant (z = 1.59, *p* = 0.056). For irradiation on the Varian accelerator, a dose-dependent relationship was found to be expressed by y = 2.64 + 17.60 × x (R^2^ = 0.99, *p* < 0.001), where y is an average of foci numbers in a cell nucleus and x is an irradiation dose (Gy). The slope coefficient was 1.27 times lower (z = 2.82, *p* = 0.002) as compared to irradiation on the AREAL accelerator with 20 Hz repetition rate.

Dose-dependent changes in 53BP1 foci numbers are presented in Figure 1b. In overall, dose–effect relationships are similar to those revealed for γH2AX foci and were found to be described by the following equations:

(1) Irradiation on the AREAL accelerator at a 20 Hz repetition rate—y = 4.81 + 20.82 × x (R^2^ = 0.97, *p* = 0.002), where y is an average of foci numbers in a cell nucleus and x is an irradiation dose (Gy);

(2) Irradiation on the AREAL accelerator at 2 Hz—y = 4.19 + 18.19 × x (R^2^ = 0.99, *p* < 0.001), where y is an average of foci numbers in a cell nucleus and x is an irradiation dose (Gy);

(3) Irradiation on the Varian accelerator—y = 3.62 + 16.35 × x (R^2^ = 0.99, *p* < 0.001), where y is an average of foci numbers in a cell nucleus and x is an irradiation dose (Gy).

No statistically significant differences between the linear slope coefficients were found after irradiation on the AREAL accelerator at two repetition rates (z = 1.14, *p* = 0.156). However, significant differences were observed between the irradiation effects on AREAL at 20 Hz and Varian accelerators, and particularly the linear slope coefficient differed by about 1.27 times (z = 1.96, *p* = 0.024).

Thus, the comparative analysis of dose-dependent changes in γH2AX and 53BP1 foci numbers after irradiation on AREAL and Varian accelerators revealed, that overall quantitative effects per dose unit are comparable. However, differences related to the changes of pulse repetition and associated dose rate used on AREAL accelerator were noted, and particularly, it was shown that decreasing the repetition rate from 20 to 2 Hz, respectively, resulted in a decrease in foci formation per dose unit by about 13–14%.

### 2.2. Post-Irradiation Changes of γH2AX and 53BP1 Foci Numbers

Then, we studied the post-irradiation changes in γH2AX and 53BP1 foci numbers in response to 1 Gy irradiation. The decrease in the time kinetics of γH2AX and 53BP foci numbers reflects the speed by which radiation-induced DSBs are repaired. The 1, 4 and 24 h after irradiation were selected as the time points.

**Figure 2:**
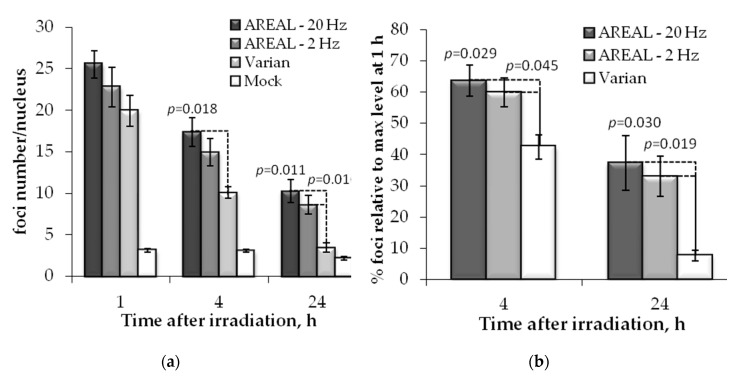
Post-irradiation changes of the γH2AX foci in HeLa cells, irradiated on AREAL (ultrashort pulsed 3.6 MeV electron beams) and Varian (quasi-continuous 4.0 MeV electron beams) accelerators at a 1000 mGy dose: (**a**) number of foci in a cell nucleus; (**b**) foci % in relation to the value at 1 h post-irradiation. Mean values derived from at least three independent experiments are shown. Error bars show SE. Statistical significance was tested using the Student *t*-test. *p* values < 0.05 are shown.

After exposure with pulsed and quasi-continuous irradiations, a significant difference in the time course of γH2AX foci number changes were observed (Figure 2). A statistically significant increase in γH2AX foci numbers at 4 h post-irradiation on the AREAL accelerator at a 20 Hz repetition rate was found compared to the irradiation on Varian. Moreover, 24 h post-irradiation, the statistically significant difference between the effects of pulsed and quasi-continuous irradiations was observed at both pulse rates (2 and 20 Hz).

Difference in the γH2AX foci numbers were observed also at 1 h after pulse and quasi-continuous irradiations, however, it did not reach statistical significance. To quantify for this trend, we calculated the relative changes in foci numbers with respect to the values at 1 h post-irradiation. It was shown that 4 h after exposure to pulsed irradiation, the relative foci number was 1.5–1.7 times higher as compared to the quasi-continuous one, while 24 h post-irradiation this value was 2.5–2.9 times higher (Figure 2b). At the same time, no significant difference between the effects of pulsed irradiation at 2 and 20 Hz was noted.

**Figure 3:**
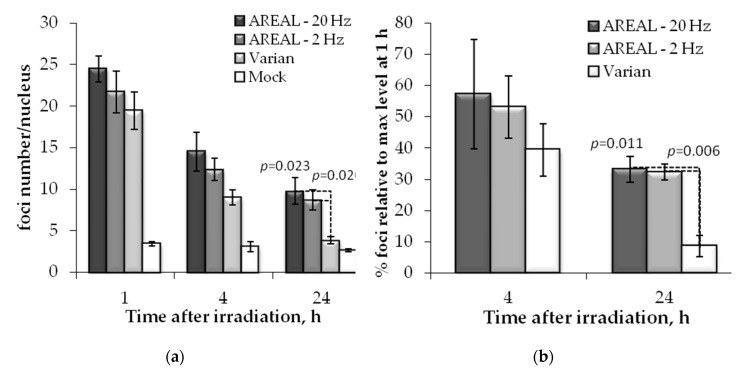
Post-irradiation changes of the 53BP1 foci in Hela cells, irradiated on AREAL (ultrashort pulsed 3.6 MeV electron beams) and Varian (quasi-continuous 4.0 MeV electron beams) accelerators at a 1000 mGy dose: (**a**) number of foci in a cell nucleus; (**b**) foci % in relation to the value at 1 h post-irradiation. Mean values derived from at least three independent experiments are shown. Error bars show SE. Statistical significance was tested using the Student *t*-test. *p* values < 0.05 are shown.

Similar trends were observed also for 53BP1 foci (Figure 3). However, in this case, a statistically significant difference in residual foci numbers was found only 24 h post-irradiation, after exposure with pulsed and quasi-continuous irradiations. At 24 h post-irradiation, the relative foci number, induced by exposure to pulsed irradiation, was about 2.3–2.5 times higher than that induced by quasi-continuous irradiation. No difference was found between the effects of pulsed irradiation at 2 and 20 Hz.

### 2.3. Dose-Dependent Changes in γH2AX Residual Foci Numbers

**Figure 4:**
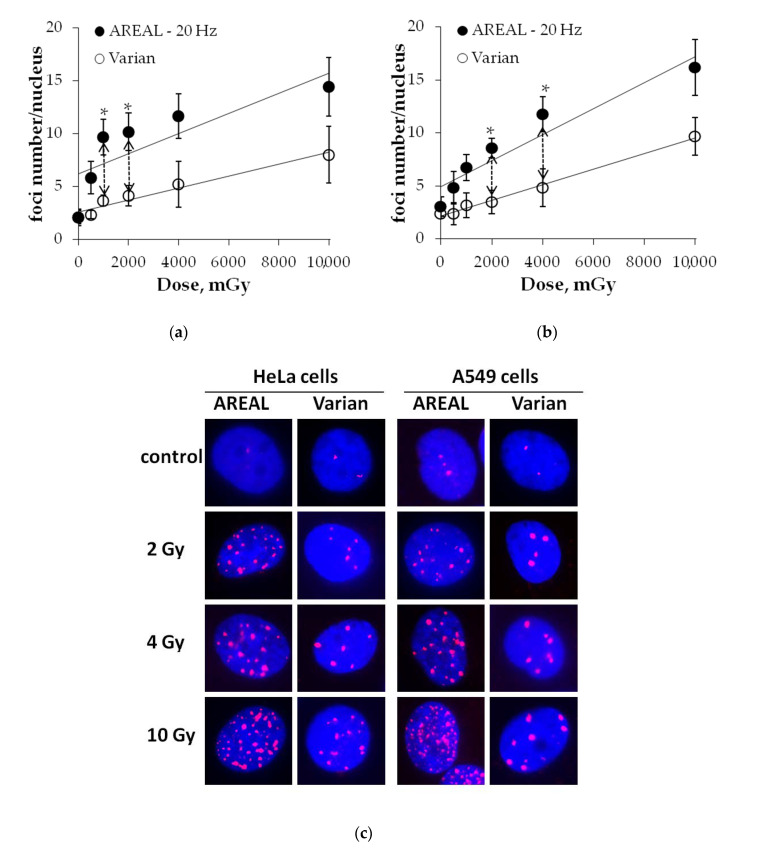
Dose-dependent changes in the γH2AX residual foci numbers in HeLa (**a**) and A549 (**b**) cells at 24 h post-irradiation by AREAL (ultrashort pulsed 3.6 MeV electron beams) and Varian (quasi-continuous 4.0 MeV electron beams) accelerators; (**c**) representative images of residual γH2AX foci in cell nuclei. Nuclei were counter-stained with DAPI, shown in blue. γH2AX foci are shown in red. Mean values derived from at least three independent experiments are shown. Error bars show SE. Statistical significance was tested using the Student *t*-test. * *p* value < 0.05.

Experiments for the dose-dependence studies of the number of residual γH2AX foci in HeLa and A549 cells at 24 h after pulse and quasi-continuous irradiations at doses of 500, 1000, 2000, 4000 and 10,000 mGy have been performed to confirm the phenomenon of the delayed elimination of DNA DSB repair proteins foci. It was shown that over the entire studied dose range, the residual γH2AX foci after exposure to the pulse irradiation was 1.7–2.9 times higher than that after quasi-continuous irradiation exposure (Figure 4). However, due to the high scatter of experimental data, the statistically significant difference was observed only after irradiation at doses of 1 and 2 Gy in the case of Hela cells (Figure 4a) and at doses of 2 and 4 Gy in the case of A549 cells (Figure 4b). It is noteworthy that after the quasi-continuous irradiation of dose-dependent relationships, changes in the residual foci number are described by linear equations for both HeLa and A549 cells (Figure 4), whereas after pulse irradiation, the linear dose-dependence of changes of residual foci number was observed only in A549 cells (y = 4.93 + 1.23 × x (R^2^ = 0.91, *p* = 0.003), where y is an average of foci numbers in a cell nucleus and x is an irradiation dose (Gy)). In the case of HeLa cells, the number of residual foci was changed insignificantly at the dose range of 1–10 Gy and the “saturation” effect was observed (Figure 4a).

The comparative analysis of the slope coefficients of dose–response changes in residual foci number has been performed in A549 cells. The linear slope coefficient was shown to be 1.7 times higher in the case of pulse irradiation compared to quasi-continuous irradiation (z = 2.5, *p* = 0.006).

Overall, the obtained results confirm the conclusion about the delayed nature of the elimination of pulse radiation-induced DSBs repair proteins foci in cancer cells compared to that after quasi-continuous irradiation.

## 3. Discussion

In this study, it was shown that exposure with both ultrashort pulsed and quasi-continuous electron irradiation at 1 h post-irradiation led to the linear dose-dependent formation of γH2AX and 53BP1 foci, which are markers of DNA DSBs. This time-point was selected since it has been reported that the maximum number of γH2AX and 53BP1 foci is formed at 0.5–1 h post-irradiation [23,24]. For quasi-continuous electron irradiation, the quantitative yields of γH2AX and 53BP1 per Gy/cell are comparable to those reported in the literature for the exposures of cancer cells with low linear energy transfer (LET) radiation (17.6 and 16.4 foci/Gy/cell, respectively) [22,24]. After exposure with ultrashort pulsed irradiation at 2 and 20 Hz, the quantitative yield of foci was higher (about 1.1 and 1.3 times), though statistically significant changes were observed only at 20 Hz.

It is important to note that the quantitative yield of DNA DSBs marker proteins foci at certain time points after irradiation depends not only on irradiation-induced initial number of DSBs, but also on the complexity of these lesions. According to the two-lesion kinetic model of double-strand breaks rejoining, DNA DSBs can be divided into simple and complex ones [25]. The repair of simple, i.e., single DSBs, is carried out very fast (15 min repair half-time), while the repair of complex DSBs, i.e., complicated by other lesions, performed slowly (10 to 15 h repair half-time) [25]. Depending on the ratio of simple and complex DSBs, the kinetics of DNA repair and accordingly, the time course of changes in DSBs foci number will vary.

In overall, the post-irradiation kinetics of change in γH2AX and 53BP1 numbers upon exposure to quasi-continuous radiation was in accordance to those reported in the literature for low LET radiation [22,24]. At 4 h post-irradiation about 45% of the γH2AX and 40% of 53BP1 foci of those observed at 1 h still were found, while at 24 h these values were 7 and 6%, respectively. Quite a different picture was shown after the exposure to ultrashort pulse radiation. At 4 h post-irradiation, about 59–64% of γH2AX and 53–57% 53BP1 foci of those observed at 1 h post-irradiation were found. It should be pointed out that though foci numbers were slightly higher after irradiation at 20 Hz, statistically significant differences induced by 2 and 20 Hz frequencies were not observed.

In addition, the dose-dependent changes in the number of residual γH2AX foci have been studied in HeLa and A549 cells at 24 h post-irradiation at doses of 500, 1000, 2000, 4000 and 10,000 mGy. It was shown that the number of residual γH2AX foci after exposure to the pulse irradiation was 1.7–2.9 times higher compared to the quasi-continuous irradiation across the entire dose range studied.

In general, the obtained data indicated slower DSB repair rate induced by ultrashort pulsed irradiation, compared to the ones induced by quasi-continuous irradiation. The pulse duration of ultrashort irradiation is only 0.4 × 10^−12^ s, however, a huge peak dose-rate of 1.6 × 10^10^ Gy/s per pulse is achieved during the pulse. Apparently, it increases the possibility of complex difficulty repairable DSBs formation. Further detailed studies of the physicochemical mechanisms of biological effects induced by sub-picosecond pulse irradiation are needed.

## 4. Materials and Methods

### 4.1. Cell Culture

HeLa (human cervical cancer cells) and A549 (human lung carcinoma) cell lines were obtained from the American Type Culture Collection (ATCC) and maintained in DMEM/F12 (Gibco, Thermo Fisher Scientific, Waltham, MA, USA) with 2.5 mM L-Glutamine (Thermo Fisher Scientific, Waltham, MA, USA), supplemented with 10% fetal bovine serum (Thermo Fisher Scientific, Waltham, MA, USA), 100 IU/mL penicillin (Sigma Aldrich, Darmstadt, Germany), and 100 μg/mL streptomycin (Sigma Aldrich, Darmstadt, Germany) at 37 °C in 5% CO_2_.

Prior to irradiation, the cells were seeded at a density of 0.4 × 10^5^ cells/mL in 2.5 mL of culture medium onto coverslips (Thermo Fisher Scientific, Waltham, MA, USA) placed inside 35 mm Petri dishes (Corning, New York, NY, USA) and incubated at 37 °C and 5% CO_2_ for 20 h.

### 4.2. Irradiation

#### 4.2.1. Ultrashort Beam Irradiation

Ultrashort beam irradiation was carried out using an electron beam generated by a laser-driven radiofrequency gun-based linear AREAL accelerator. The characteristics of the AREAL accelerator have been described previously [26]. The parameters of the AREAL laser-generated electron beam are presented in Table 1.

The dosimetric measurements were performed with a Faraday cup (commercially available), estimating the integral dose over the pulse. Cells were irradiated at doses of 0.25–10 Gy (~140 electron pulses per 1 Gy) with a repetition rate of 2 or 20 Hz. A peak dose rate of 1.6 × 10^10^ Gy/s was estimated from the electron pulse duration of 4.5 × 10^–13^ s, based on the laser pulse length, acceleration process, and electron beam transport. The mean absorbed dose-rate of 1.17 ± 0.02 Gy/min (repetition rate 2 Hz sample mass of 4.2 g) and 11.70 ± 0.98 Gy/min (repetition rate 20 Hz sample mass of 4.2 g) was calculated over the period of irradiation and 1% charge fluctuation and 1% beam energy fluctuation was taken into account.

#### 4.2.2. Quasi-Continuous Irradiation

Quasi-continuous irradiation was carried out using the Varian Trilogy (Varian Medical Systems, Palo Alto, CA, USA) electron linear accelerator. Characteristics: electron energy 4.0 MeV, dose-rate 5.6 Gy/min, electron beam size 250 × 250 mm. Dosimetry was performed by ionization method in a water phantom according to the International Atomic Energy Agency (IAEA) TRS-398 international protocol [27].

### 4.3. Immunofluorescence Staining

Cells were fixed on coverslips in 2% paraformaldehyde in PBS (pH 7.4) for 20 min at room temperature, followed by two rinses in PBS and permeabilization in 0.3% Triton-X100 (in PBS, pH 7.4), supplemented with 2% bovine serum albumin (BSA) to block nonspecific antibody binding. Cells were incubated for 1 h at room temperature with primary antibody against γH2AX (dilution 1:200, clone EP854(2)Y, MerckMillipore, Burlington, VT, USA) and 53BP1 (dilution 1:200, clone BP13, Merck-Millipore, Burlington, VT, USA), diluted in PBS with 1% BSA. Then the specimens were washed three times with PBS (pH 7.4) and incubated for 1 h at room temperature with secondary IgG (H + L) goat anti-mouse (Alexa Fluor 488 conjugated, dilution 1:600; Merck-Millipore, Burlington, VT, USA) and goat anti-rabbit (rhodamine conjugated, dilution 1:400; Merck-Millipore, Burlington, VT, USA) diluted in PBS (pH 7.4) with 1% BSA. The coverslips were then rinsed several times with PBS and mounted on microscope slides with ProLong Gold medium (Life Technologies, Carlsbad, SA, USA) with 4′,6-diamidino-2-phenylindole (DAPI) for DNA counter-staining. Imaging of immunocytochemical specimens were performed using a Nikon Eclipse Ni-U fluorescent microscope (Nikon, Tokyo, Japan) equipped with a ProgRes MFcool high-resolution video camera (Jenoptik AG, Jena, Germany). The filter sets: UV-2E/C (340–380 nm excitation and 435–485 nm emission), B-2E/C (465–495 nm excitation and 515–555 nm emission), and Y-2E/C (540–580 nm excitation and 600–660 nm emission). Between 300 and 400 cells were imaged for each data point. Foci were enumerated using the FociCounter software (http://focicounter.sourceforge.net/).

### 4.4. Statistical Analysis

Statistical and mathematical analyses of the data were conducted using the Statistica 8.0 software (StatSoft, Tulsa, OK, USA). The results are presented as the means of three independent experiments ± standard error. Statistical significance was tested using the Student *t*-test. The slope coefficients of dose–response curves were compared using the Z-test.

## Figures and Tables

**Table 1:** Characterization of the AREAL laser-generated electron beam.

AREAL Beam Parameters		UV Laser Parameters	
Beam charge (pC)	30	Wavelength (nm)	258
Electron energy (MeV)	3.6	Pulse energy (μJ)	200
Pulse duration (fs)	450	Repetition rate (Hz)	1–50
Pulse repetition rate (Hz)	1–50	Energy stability	<1%
Beam spot size (mm)	15	Beam divergence (mrad)	<0.3
Norm. emittance (mm-mrad)	<0.5	Beam diameter (mm)	2.0
RMS energy spread	<1.15%	-	-
Online dose information	Faraday cup	-	-
